# Fitness changes in wild soybean caused by gene flow from genetically modified soybean

**DOI:** 10.1186/s12870-023-04398-2

**Published:** 2023-09-14

**Authors:** Li Zhang, Laipan Liu, Zhixiang Fang, Wenjing Shen, Ying Dai, Ruizong Jia, Jingang Liang, Biao Liu

**Affiliations:** 1 Key Laboratory on Biosafety of Nanjing Institute of Environmental Sciences, Ministry of Ecology and Environment, Nanjing, 210042 China; 2 State Environmental Protection Scientific Observation and Research Station for Ecology and Environment of Wuyi Mountains, Nanjing Institute of Environmental Sciences, Ministry of Ecology and Environment, Nanjing, China; 3 Hainan Key Laboratory for Biosafety Monitoring and Molecular Breeding in off-Season Reproduction Regions, Sanya, China; 4 Development Center of Science and Technology, Ministry of Agriculture and Rural Affairs, Beijing, 100176 China

**Keywords:** Transgenic soybean, Gene flow, Hybrid fitness, Ecological risk

## Abstract

**Background:**

Crop-wild hybridization has generated great concerns since gene flow can be an avenue for transgene escape. However, a rather limited number of studies on risk assessment regarding the dispersion of transgenes from GM soybean to populations of its wild relatives have been previously conducted.

**Results:**

The results of the 3-year experiment demonstrated that hybrids between GM soybeans and wild soybean had lower seed germination and higher seed productivity than GM soybean. Both of these features of hybrid (especially F_2_ and F_3_) were similar to those of wild soybean. Furthermore, the foreign protein was stably expressed in hybrid EPSPS positive plants; however, no difference was observed in agronomic measurements between hybrids that are glyphosate sensitive or resistant, homozygous or heterozygous for the transgene, indicating that the presence of the *EPSPS* transgene does not affect the vigor of hybrid. In contrast, hybridization between GM soybean and wild soybean may have more impact on hybrid growth and fecundity, this increase in biomass and yield confers a potential competition benefit to hybrids.

**Conclusions:**

Gene flow from GM soybean to wild soybean has the potential to promote the adaptability of hybrids and may increase the possibility of dispersal of transgenes in wild soybean relatives.

**Supplementary Information:**

The online version contains supplementary material available at 10.1186/s12870-023-04398-2.

## Background

Pollen-mediated gene flow between a genetically modified (GM) crop and its wild relatives has generated great concerns because transgene escape can lead to unpredictable ecological risks [1–4]. By means of gene flow, a transgene has the potential to transfer from a GM crop to populations of its wild relative and endure or spread within the wild population via subsequent hybridization and introgression between the GM crop and its wild relative [5]. This may have the potential to exacerbate weed problems by providing novel traits that allow these plants to compete better, produce more seeds, and become more abundant [6], resulting in changing variations in wild populations [7]. Therefore, determining the ability of hybrid offspring to survive and reproduce in their new environment [8–11] has gradually become one of the effective models to predict consequences of gene flow from GM crops to wild relatives [12–14].

As the largest transgenic crop species in terms of planting area worldwide, GM soybean has been widely commercially adopted in many countries, such as the United States, Brazil and Argentina. However, unlike these countries, China has not yet implemented GM soybean commercially. One of the major reasons for this is the environmental concerns about the gene flow from GM soybean to their wild relatives. Annual wild soybean (*Glycine soja*), an annual, self-pollinating plant species, is widely distributed in Japan, Korea and northeastern Russia, and China is one of the main distribution areas of wild soybean. Different from cultivated soybeans, wild soybean exhibits several unique traits, such as vine stems, pod shattering, blooms and small hard seeds [15]. In addition, the species has excellent characteristics, such as high protein, high yield, and tolerance to salt stress [16], which are valuable genetic resources for cultivated soybean breeding. Since wild soybean chromosome number is the same as that of cultivated soybean (2n = 40), outcrossing between them can frequently occur under natural field conditions; some studies have shown that gene flow between cultivated and wild soybean occurs at very low frequencies [17–19]. However, wild soybean commonly grows throughout almost all of China, and their distributions largely overlap with the distributions of cultivated soybean fields, especially in northeastern and southeastern China [20]. While more favorable conditions, such as flowering synchrony and certain climatic conditions, are available, greater gene flow may be observed [21].

At present, a few studies have been conducted to evaluate gene flow and the growth performance of hybrids between GM soybean and wild soybean under greenhouse or field conditions. Kan et al. [22] measured the F_1_ and F_2_ hybrids of four wild soybeans and glyphosate-resistant soybean in a greenhouse and found that hybrids had similar pod and seed numbers per plant as their wild relatives. Field experimental studies conducted by Yook et al. [21] and Guan et al. [23] also found that hybrids (especially F_2_ hybrids) showed similar characteristics to wild soybean in vegetative growth and seed productivity, and the results of previous studies [21–23] indicate that hybrids with a higher fitness level might be associated with a higher ability to adapt to the environment and may therefore be beneficial for establishing transgenes in populations.

The expression of endogenous genes such as *Bt* and *CP4-EPSPS* could improve resistance to insects or herbicides; if the transgene is normally expressed in crop-wild hybrids and progenies and inherited between different generations, the transgene may change a certain trait of wild plants, possibly leading to further undesired environmental consequences [5]. Therefore, to evaluate the risk of GM soybean and its hybrids resulting from gene flow, it is necessary to investigate protein expression data for assessing and monitoring the biosafety of GM crops and hybrids; however, previous studies mainly focused on vegetative and reproductive hybrids [21–23], and the protein levels in hybrid plants were not investigated. In addition, wild soybean seeds have strong physical dormancy, while cultivated soybean seeds do not [24]. The seed dormancy of the progeny of the hybrid obtained from a cross of wild soybean and GM soybean is still not clear, especially in higher hybrid generations, such as the F_2_ and F_3_ generations. The hybrid populations will segregate as homozygous resistant plants (RR), heterozygous resistant plants (RS) and homozygous susceptible plants (SS) based on endogenous genes, and the seed dormancy, vegetative growth and fecundity of these three groups are unknown.

China has always attached great importance to the application of GM technology to improve agricultural productivity. After over 20 years of development, GM soybean in China is now closer to the commercialization stage. It is now necessary to monitor the possible gene transfer from GM crops to wild soybean and investigate the characterization of such hybrids before large-scale commercial production of GM soybean. Therefore, the present study was carried out to measure seed dormancy and plant performance of hybrids between GM soybean and wild soybean under greenhouse conditions, to better understand potential weed risk of transgene escape from GM soybean to its wild relative.

## Results

### Genotyping assay for F_2_ and F_3_ generations

QPCR and dPCR assays were used to identify RR, RS and SS genotypes of the F_2_ and F_3_ populations, a total of 168 F_2_ and 123 F_3_ populations were submitted to genotype analysis. In this research, the identification and screening of 168 F_2_ individuals showed that the F_2_ population had 45 RR, 86 RS and 37 SS plants, and the frequency of the *EPSPS* genotype showed a 1:2:1 genetic ratio (χ²=0.857, df = 2, p = 0.651, Chi-square test). Among the 123 F_3_ population, 38 plants were genotyped as RR, 63 RS, and the remaining 22 SS and F_3_ populations were shown to segregate at a ratio of 1:2.86:1.73. The genotype test results are shown in Additional file 1 and Additional file 2.

### Seed germination and vitality of ungerminated seeds

**Fig. 1:**
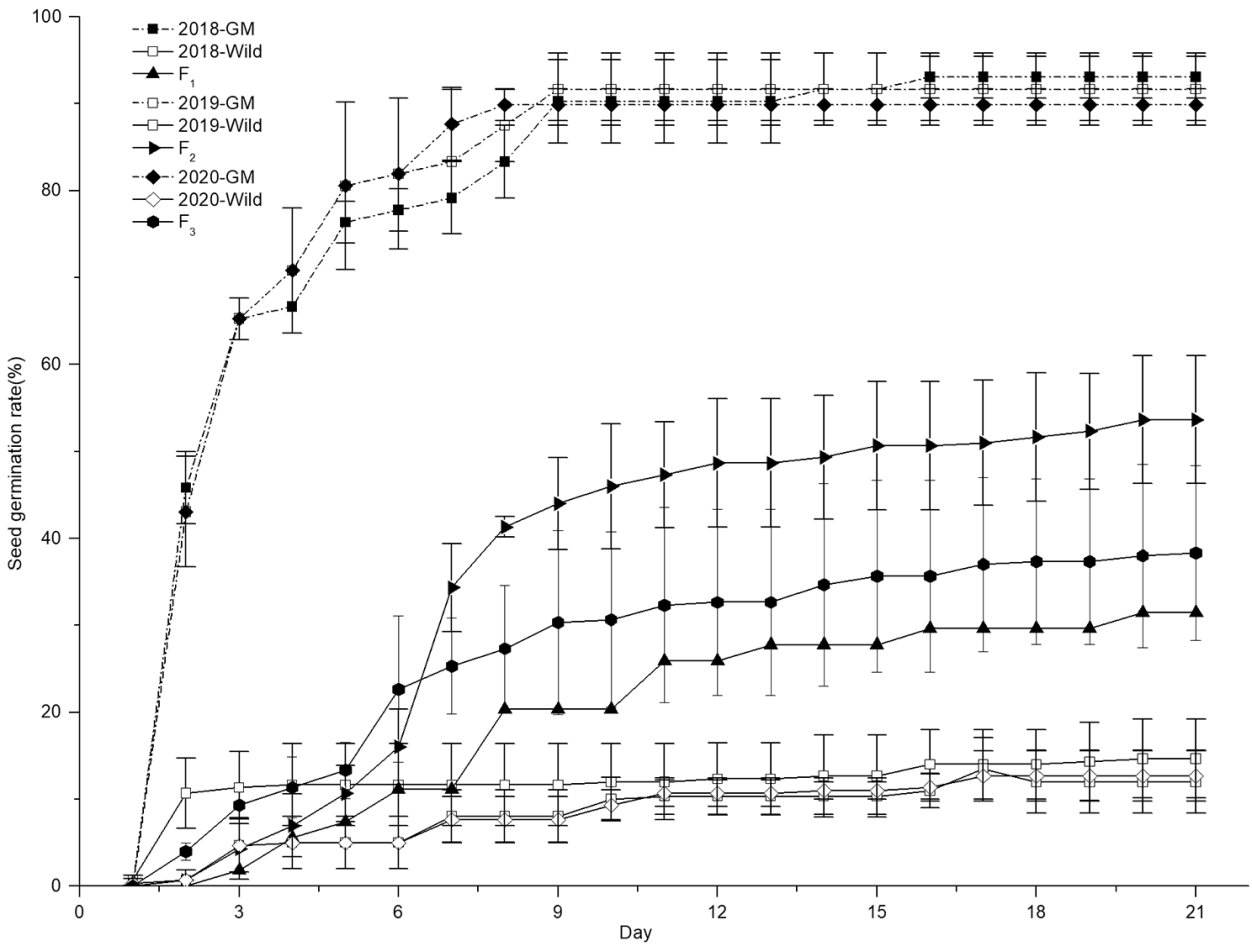
Seed germination rate of GM, wild, and hybrid soybeans. Error bars indicate standard error

The 21-day seed germination of GM soybean, wild soybean, and hybrids are shown in Fig. 1. In 2018, GM soybean had the highest total germination rate (93.05%), while wild soybean had the lowest germination rate (14.67%). The F_1_ hybrid germination rate (31.48%) was intermediate between that of wild and GM soybeans, and there were significant differences in seed germination between the F_1_ hybrid and parental lines.

The germination of GM and wild soybean was similar in different years, and the germination of F_2_ and F_3_ hybrids was 53.67% and 38.33%, respectively. Both F_2_ and F_3_ hybrids exhibited intermediate germination between wild and GM soybeans, and significant differences were observed in seed germination between F_2_ and F_3_ hybrids and the parental lines.

At the end of the germination test, all non-germinated GM seeds were found to be mildewed and rotten; in contrast, most ungerminated wild soybean, F_1_, F_2_, F_3_ seeds seemed normal and did not change in shape, size or color. These seeds were evaluated for their ability to germinate after partial seed coat removal, and the results showed that the five-day seed germination of all observed wild soybean and hybrids was above 87.5% (Table 1).

**Table 1:** Germination rate of ungerminated seeds after the seed coat was manually removed

Year	Material	Seed no. used for the germination test	Average no. of germinated seeds	Germination rate (%)
2018	Wild	70 ± 0	68 ± 2	97.14 ± 2.86
	F_1_	32	28	87.5
2019	Wild	70 ± 0	65 ± 3	93.33 ± 3.60
	F_2_	39 ± 9	35 ± 8	89.08 ± 2.18
2020	Wild	70 ± 0	67 ± 4	95.24 ± 5.95
	F_3_	48 ± 4	44 ± 4	91.67 ± 3.22

Note: Data are presented as means ± SD.

### Aboveground biomass

**Fig. 2:**
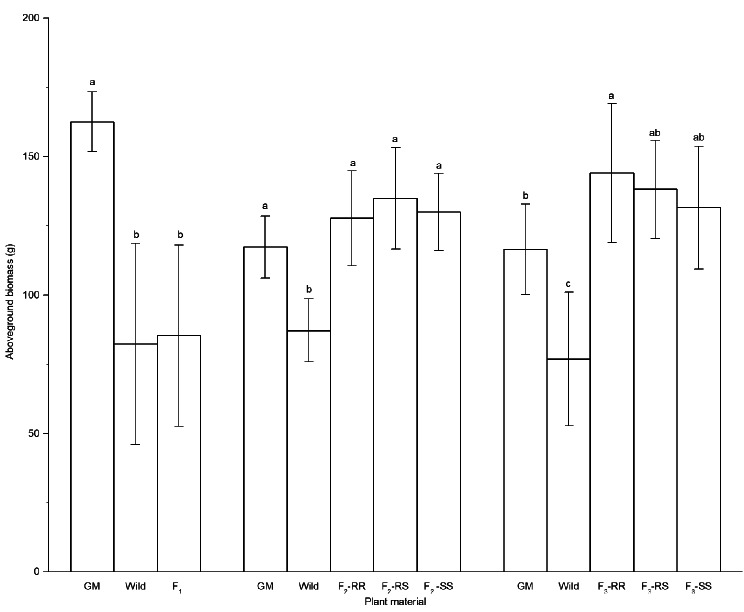
Aboveground biomass of GM, wild, and hybrid soybeans. *GM* genetically modified, *RR* homozygous resistant, *RS* heterozygous resistant, *SS* homozygous susceptible. Different lowercase letters denote statistical differences between treatment groups at the 5% level according to Tukey’s test

The average aboveground biomass of the F_1_ plants and their hybrid female parent, wild soybean, was not significantly different, 85.35 g versus 82.28 g, respectively. In contrast, the average aboveground biomass of GM plants was significantly higher than that of F_1_ and wild soybean (Fig. 2).

The aboveground biomass of the F_2_ population and GM soybean was significantly higher than that of wild soybean, and there were no significant differences between the F_2_ population and GM soybean in aboveground biomass. Our results also did not show any significant difference among the F_2_ population in aboveground biomass.

The total aboveground biomass of the F_3_ population ranged from 144.06 g, 138.18 g and 131.54 g for the RR, RS and SS plants, respectively, and no significant differences were found in biomass within the F_3_ population. However, F_3_ had a significantly higher aboveground biomass (*P* < 0.05) than wild soybean, while F_3_ had slightly higher aboveground biomass than GM soybean, but the difference was not significant.

### Fecundity

The pod number per plant of wild soybean and F_1_ hybrids was significantly higher than that of GM soybean in 2018. Wild soybean produced 565 pods per plant, while F_1_ hybrids produced 353 pods, and a significant difference was observed in the wild soybean and F_1_ hybrid groups (Table 2).

F_2_ homozygous resistant, heterozygous and homozygous susceptible plants produced 561, 545, and 686 pods per plant, respectively, and no significant differences were found between RR, RS and SS. Analysis of variance also showed that the pod numbers were not significantly different between wild soybean and F_2_ hybrids (Table 2).

F_3_ hybrids produced a much larger number of pods than GM soybean, and the number was slightly lower than that of wild soybean; however, no significant differences were observed between the two groups. In addition, pod numbers were compared between different genotypes of the F_3_ population, and no significant of the parameters differed among RR, RS, and SS.

The seed number and full seed number per plant for wild, GM and hybrid soybeans are shown in Table 2. The seed number and full seed number of wild soybean and the hybrids were significantly higher than those of GM soybean. F_1_ hybrids produced 491 seeds and 426 full seeds, which were significantly different from wild soybean, while F_2_ and F_3_ hybrids had a similar number of seeds as wild soybean.

In three years, GM soybeans had higher 100-seed weights than wild and hybrid soybeans. The F_1_ hybrid 100-seed weight was more similar to that of wild soybean, no differences were recorded for 100-seed weight between the F_1_ hybrid and wild soybean, however, both F_2_ and F_3_ hybrids had higher 100-seed weights than their wild soybean counterparts; no parameters significantly differed among RR, RS, and SS in the F_2_ or F_3_ population.

**Table 2:** Fecundity of GM, wild, and hybrid soybeans

Year	Material	No. of pods per plant	No. of seeds per plant	No. of full seeds per plant	100-seed weight (g)
2018	GM	122 ± 43c	234 ± 76b	189 ± 66b	17.62 ± 2.85a
	Wild	565 ± 203a	1138 ± 406a	1010 ± 400a	1.91 ± 0.04b
	F_1_	353 ± 136b	491 ± 190b	426 ± 174b	1.81 ± 0.22b
2019	GM	116 ± 33b	222 ± 58b	199 ± 54b	18.80 ± 2.00a
	Wild	631 ± 136a	1099 ± 219a	949 ± 230a	1.89 ± 0.05c
	F_2_-RR	561 ± 179a	881 ± 274a	829 ± 258a	4.93 ± 0.33b
	F_2_-RS	545 ± 122a	923 ± 287a	869 ± 277a	4.81 ± 0.63b
	F_2_-SS	686 ± 123a	1009 ± 231a	953 ± 208a	4.47 ± 0.34b
2020	GM	115 ± 40b	252 ± 76b	217 ± 69b	15.34 ± 0.74a
	Wild	564 ± 144a	1122 ± 225a	944 ± 406a	1.98 ± 0.12c
	F_3_-RR	549 ± 152a	1053 ± 305a	995 ± 322a	4.95 ± 0.31b
	F_3_-RS	524 ± 113a	985 ± 261a	931 ± 251a	4.19 ± 0.26b
	F_3_-SS	527 ± 158a	1008 ± 271a	951 ± 273a	4.02 ± 0.21b

Note: Data presented are means ± SD, means with different superscripts in the same column are significantly different (Tukey’s test, *P* < 0.05). *GM* genetically modified, *RR* homozygous resistant, *RS* heterozygous resistant, *SS* homozygous susceptible

### CP4-EPSPS protein expression levels in samples

**Fig. 3:**
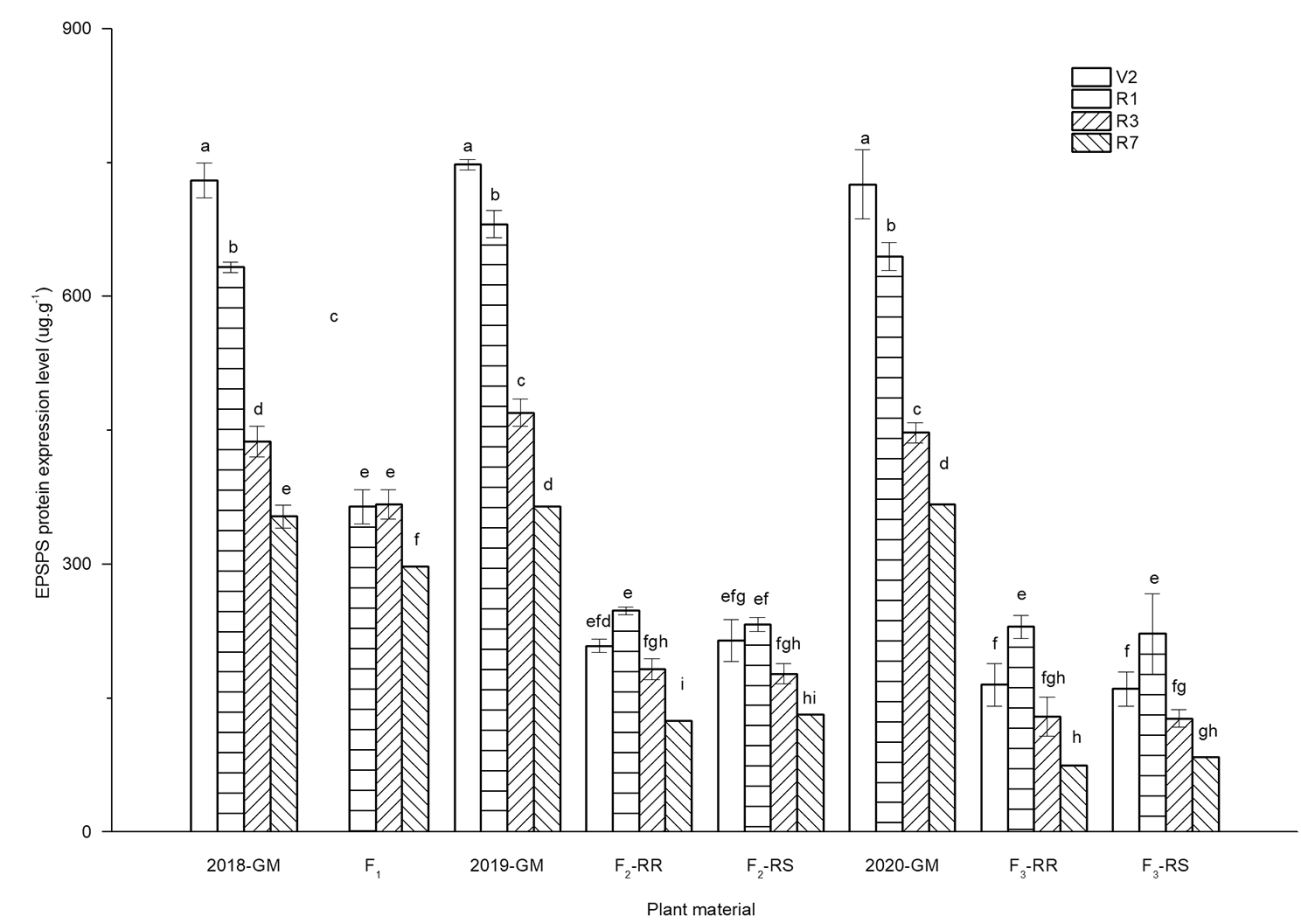
ELISA detection of CP4-EPSPS protein in GM and hybrid soybeans. *GM* genetically modified, *RR* homozygous resistant, *RS* heterozygous resistant. Different lowercase letters denote statistical differences between treatment groups at the 5% level according to Tukey’s test

The expression levels of the *CP4*-*EPSPS* gene in plant leaf samples were assessed during different growth stages of soybean. The results showed that all wild soybean samples and SS plants were negative for EPSPS expression. In contrast, EPSPS was detectable at different stages in F_1_, F_2_ RR, F_2_ RS, F_3_ RR, F_3_ RS, and GM plants, and the protein levels in both hybrids and GM soybean were influenced by growth stages. The expression of EPSPS declined significantly as plants matured (Fig. 3). The levels of EPSPS in GM plants ranged between 364.28 and 747.79 µg/g; in F_2_ plants, they ranged between 124.15 and 247.89 µg/g, and the protein levels were highest in the R2 stage (230.07 µg/g) and lowest in the R7 stage (74.29 µg/g) in F_3_. In addition, similar levels of relative EPSPS expression were observed for F_2_ and F_3_ RR and RS plants, and there were no significant differences between RR and RS in different plant generations.

## Discussion

Crop-wild/weed hybridization has generated great concerns simply because gene flow can be an avenue for transgene escape, which could alter the genetic make-up of both populations [25, 26]. Evaluation of the fitness of crop-wild hybrids and their parents, especially the wild parents, is a direct way to investigate the potential consequences of crop-to-wild gene flow [27]. In this study, agronomic comparisons were evaluated among GM soybean, wild soybean and their F_1_, F_2_ and F_3_ progenies, with implications for transgene escape from GM soybean varieties, resulting in a better understanding of its consequences.

### Seed dormancy

Seed dormancy is an important component of plant fitness that causes a delay in germination until the arrival of a favorable growth season [28]. Wild soybean has strong physical seed dormancy, which is mainly caused by the physical structure of the seed coat; the seed usually does not permit the imbibition of water immediately after immersion [29], and the high seed dormancy of wild soybean can allow the seed to remain viable for long periods in the soil and delays seed germination until environmental conditions are correct [20]. In contrast, *G. max* seeds undergo little to no dormancy because high germination is important for soybean cultivation and food processing. If seeds of hybrids between wild and GM soybean could have stronger dormancy like their wild relatives, it may favor the formation of a longer-lived seed bank enriched with the transgenic seeds.

In the present study, 21-day seed germination results showed that, although F_1_, F_2_ and F_3_ hybrid seed germination was significantly higher than that of wild soybean, about half of the F_1_, F_2_ and F_3_ seeds did not germinate, and most ungerminated F_1_, F_2_, F_3_ seeds were deemed normal, i.e., not changed in shaped, size or color. After their partial seed coats were removed, almost all the ungerminated seeds were viable, and the seed germination of all observed hybrid seeds was above 87.5%, suggesting that hybrid soybean had similar germination characteristics as wild soybean, and the hybrid seeds can persist for a considerable amount of time in the soil seed bank.

### Aboveground biomass

Previous studies have found that F_1_ hybrids between wild soybean and glyphosate-resistant soybean had similar dry weights compared to that of wild soybean, while both GM and non-GM F_2_ hybrids had a significantly higher aboveground biomass than wild soybean in the field conditions [23]. A similar conclusion was reported by Yook et al. [21], who found that hybrids showed similar characteristics to wild soybean in above-ground biomass. Consistent with previous research, this study also found that hybrids between wild soybean and GM soybean, especially F_2_ and F_3_, had stronger vegetative growth vigor than wild soybean, and the increased aboveground biomass is conducive to improve competitive ability, this phenomenon was also observed in *Brassica rapa* [30, 31], rice [32] and sunflower [33], and the increased growth of hybrids compared with that of wild plants might be due to the paternal parents enhancing the plant performance of the hybrid [33].

### Fecundity

F_1_, F_2_, and F_3_ hybrids mainly grew well and produced more pods and more seeds than GM soybean, and their fertility increased similarly to that of wild soybeans. Importantly, there were no significant differences in F_2_ and F_3_ RR, RS or SS plants in pod and seed numbers per plant, indicating that the *EPSPS* gene and its copy number did not significantly affect the fecundity of the hybrids. Similar to our study, in a two-year field experiment, Yook et al. [21] reported that F_1_ and F_2_ hybrids had similar seed production to that of wild soybean, and no differences were found for 100-seed weight between F_1_ hybrid and wild soybeans. However, F_2_ hybrids had a higher 100-seed weight than their wild soybean counterparts; these results are consistent with our findings. In addition, our results also showed all seed parameters were not significantly different among RR, RS and SS in the F_2_ or F_3_ population. Our findings and those of previous studies suggest that hybrids possess higher seed production potential [21–24], which could make hybrids more competitive during natural selection than their wild parents.

### CP4-EPSPS protein expression levels

To facilitate the biosafety assessment of transgene escape to populations of wild relative species, it is important to conduct scientific research to properly estimate the expression levels of transgenes in wild individuals as well as the inheritance of the transgenes in wild populations [5, 34, 35]. In the present study, the ELISA results showed that EPSPS protein was detectable in F_1−_, F_2−_ and F_3_-resistant plants, suggesting that the transgene will be able to confer tolerance to glyphosate in the new host wild population. This observation was consistent with the findings of Kubo et al. [24]. In addition, it is worth noting that a significant decline in the total protein content in both hybrids was observed compared to that in GM soybean. Since tolerance to glyphosate is very dependent on EPSPS protein expression levels in plant tissues, a reduction in the amount of endotoxin proteins in hybrids may contribute to the variability in tolerance. Consistent with our results, a similar conclusion was reported by Zhu et al. [34], who also reported a decrease in Bt protein content in transgenic *Brassica rapa* and crop-weed hybrids. This difference may be associated with a weedy genetic background, positional effects, and the number of transgenes inserted per event [30].

Our study confirmed that the CP4-EPSPS protein was stably expressed in the hybrid soybean line, endowing these hybrid soybeans with herbicide tolerance, and the RR, RS and SS of F_2_ or F_3_ populations had similar seed germination, aboveground biomass, pod and seed number per plant and 100-seed weight, which indicated that the presence and absence of *EPSPS* or the copy number of the *EPSPS* gene were not significantly correlated with hybrid vegetative growth and fecundity. In contrast, heterosis between GM and wild soybean raises new competitive advantages for hybrids, allowing hybrids to obtain some similar growth characteristics as female wild soybean, such as seed dormancy, a higher stable grain weight, and greater pod and seed numbers per plant; these growth characteristics could increase the possibility of dispersal of transgenes through seed systems and may adversely affect genetic and species diversity of wild soybean. Thus, it is critical to build effective risk management and control measures for the gene flow of transgenes from GM soybean to wild soybean before commercial planting of GM soybean in China.

## Conclusions

In conclusion, the results of the present study indicate that gene flow from GM soybean to wild soybeans may confer a survival advantage to their hybrids. Hybrids had similar germination characteristics and seed productivity as the wild parent, and these changes in agronomically important traits may lead to stronger competitive ability, resulting in rapid accumulation and spread of the transgenes in the wild soybean population. Therefore, there is a concern that large-scale planting of transgenic soybean will pose potential threats to the genetic diversity of wild soybean populations in China. In addition, while our study mainly focused on the agronomic performance of hybrids, the pod shattering trait of wild soybean was also observed in hybrids, suggesting that a considerable number of mature hybrid seeds can enter the soil via pod shattering; therefore, future studies focusing on the characteristics and fates of hybrid seeds in the soil may help determine the persistence of transgenes in soil seed banks and supplement the existing data on environmental consequences.

## Methods

### Plant materials and management

Roundup Ready (RR) soybean (GTS40-3-2, labeled GM) and wild soybean (Jiang pu) were used in this study; seeds of both varieties were kindly provided by the weeds research laboratory of Nanjing Agricultural University (Nanjing, China). The GM soybean expressing the synthetic *CP4-EPSPS* gene confer tolerance against glyphosate herbicide and has been approved in many countries around the world [36]. Since photoperiod has large effects on growth and seed yield of wild soybean, local plant populations in the Nanjing region were chosen as the research object and were obtained in Jiang pu (32.05°N, 118.62°E), Nanjing, China.

In 2017, using GM plants as the male parent (the pollen donor) and wild soybean as the female parent (the pollen recipient), crosses were performed in July by artificial pollination, and 54 F_1_ hybrid seeds were collected in mid-October. The next year (2018), 23 of 54 F_1_ seeds germinated and were subsequently transplanted, and the hybridity of F_1_ plants was confirmed by spraying with glyphosate (14.4 g/L) and PCR analysis as reported by previous studies [37]. Among these 23 F_1_ individuals, 11 plants were glyphosate resistant and were grown in a greenhouse with their parental lines to examine their characteristics and produce second filial generation (F_2_) seeds by self-pollination. F_2_ hybrids were examined for their characteristics and harvested separately to obtain third filial generation F_3_ seeds by self-pollination in 2019, and F_3_ individuals were planted to evaluate their traits in 2020.

The experiment was carried out over four consecutive years, 2017–2020, in a greenhouse at the Key Laboratory on Biosafety of Nanjing Institute of Environmental Sciences. Soybean plants were transplanted and grown in a plastic pot (730 mm × 560 mm × 230 mm) filled with mixed soil containing farmland soil and soil composite (25% peat, 25% compost, 25% perlite, 25% vermiculite) at a ratio of 1:1. A bamboo pole (diameter of 1.5 cm and height of 230 cm) was inserted into the pot and carefully fixed to allow for plant climbing. Before the pod color turned from green to brown or black, each plant was bagged loosely with a 1-mm nylon mesh to prevent seeds from splashing. During the plant growing season, weeds were manually removed from cultivation pots, and agricultural agents such as plant growth regulators, insecticides, and fertilizers were not applied.

### Genotyping assay for F_2_ and F_3_ generations

The genomic DNA of the fresh leaf samples from GM, wild, F_2_ and F_3_ plant were extracted and purified using a DNeasy® Plant Mini Kit (Qiagen, Hilden, Germany). according to the manufacturer’s instructions. The quality and quantity of the extracted DNA were determined using absorbance measurements at 260 and 280 nm wave lengths and 1% agarose gel electrophoresis respectively. High quality genomic DNA (260/280 ratio of ≥ 1.8) was used as a template for qPCR to determine the relative *EPSPS* gene copy number, and the *lectin* gene was used as an endogenous reference gene of soybean in the PCR; the sequences of the primers and probes used in this present study are shown in Table 3.

**Table 3:** Sequences of primers and probes used for the *lectin* and *EPSPS* genes

Target gene	Primer	Primer sequence(5’-3’)	Product size
*lectin*	lectin-F	GCCCTCTACTCCACCCCCA	118 bp
	lectin-R	GCCCATCTGCAAGCCTTTTT	
	lectin-P	FAM AGCTTCGCCGCTTCCTTCAACTTCAC-BHQ1	
*EPSPS*	EPSPS-Q-1 F	TTCATTCAAAATAAGATCATACATACAGGTT	84 bp
	EPSPS-Q-2R	GGCATTTGTAGGAGCCACCTT	
	EPSPS-Q-1P	FAM-CCTTTTCCATTTGGG-BHQ	

#### TaqMan real-time PCR

TaqMan real-time PCR assays were performed using an ABI 7900HT thermocycler (Applied Biosystems, United States). Each 25 µL of PCR mixture contained 12.5 h qPCR Master Mix (Huirui biotechnology, China), 0.4 µL each of 10 µM forward and reverse primers, 0.2 µL of 10 µM probe, 6.5 µL of nuclease free water, and 5 µL of test sample DNA. The cycling conditions were amended to 95 °C for 15 s, followed by 45 cycles of 95 °C for 15 s and 60 °C for 60 s. To obtain reliable results, Ct values and the ∆Ct between the Ct for the transgene and the Ct for the endogenous control were used to determine which plants contained the transgene. Samples were considered positive for amplicon production when the *lectin* gene Ct values and *EPSPS* gene Ct values were both < 35, and the amplification plot clearly demonstrated an exponential increase in the reporter signal in duplicate PCRs. A negative result was assigned when *lectin* gene Ct values were < 35 and no amplification of the *EPSPS* gene occurred. Samples with *lectin* gene Ct values < 35 and *EPSPS* gene Ct values > 35 were considered indeterminant and required repeat testing.

#### Digital PCR

Samples testing positive by real-time PCR were analyzed for *lectin* and *EPSPS* copy number by digital PCR. The digital PCR mixtures were prepared as 20 µL total volumes, which included 10 µL 2 × ddPCR Supermix (Bio-Rad Laboratories, Hercules, CA), 0.4 µL each of the forward and reverse primers, 0.2 µL probe, 1 µL DNA (20 ng/µL), and 8 µL RNase/DNase-free water. Droplets were generated using 20 uL reaction mixture and 70 uL oil with the QX200 Droplet Generator (Bio-Rad Laboratories). Droplet-partitioned samples were then transferred to a 96-well PCR plate, sealed and cycled in a T100 Thermal Cycler (Bio-Rad) under the following cycling protocol: 95 °C for 10 min (1 cycle); then 40 cycles of 95 °C for 15 s and 57.7 °C for 1 min; 98 °C for 10 min, and then held indefinitely at 4 °C. After thermal cycling, droplets were analyzed for positive and negative signals using the QX200 droplet reader (Bio-Rad Laboratories). For each digital PCR sample, the same process was performed in triplicate. Data analysis was performed when the number of droplets produced was more than 8000. The copy number ratio, which is expressed as a ratio between target and reference *lectin* genes for each DNA sample, was calculated and directly used as an indicator for identifying heterozygous and homozygous individuals. A copy number ratio close to 1 would suggest that the sample is a homozygous individual, and a copy number ratio close to 0.5 would suggest a heterozygous individual.

### Investigation of plant characteristics

#### Seed dormancy

In 2018, 300 seeds from GM and wild soybean samples were grouped into three replicates, and three replicates of 100 seeds each and all 54 F_1_ seeds were then placed individually in 12-well cell culture plates (Corning Costar, New York, USA) with two layers of filter paper. Finally, 400 µL sterile distilled water was added to each well, and the plates were kept in climate chambers (Binder model KBF 720, Tuttlingen, Germany) under 55% RH, 25 ± 2 °C and continuous dark conditions for 21 days. The number of germinated seeds per day was recorded (i.e., radicle protrusion > 5 mm) and was expressed as a percentage of the total number of tested seeds (germination percentage). The germinated seeds were removed immediately once they were counted to prevent any counting errors. Germination experiments of GM soybean, wild soybean, hybrid F_2_ and hybrid F_3_ seeds were carried out under the same experimental conditions in 2019 and 2020.

At the end of the germination test, all ungerminated seeds were carefully collected and dried again at 25 °C to evaluate their ability to germinate. All normal-shaped seeds were selected, partial seed coats were removed by scraping a small portion of the seed coat with a knife, and seed germination was examined as previously described.

#### Aboveground biomass

At maturity, 10 plants of each material were randomly selected and then naturally air-dried for one week, and dry weight was recorded using a balance (PB602-N, Mettler Toledo, Nänikon-Uster, Switzerland).

#### Fecundity

Ten soybean plants were selected from each genotype at random for recording the number of pods per plant, total number of seeds per plant (number of seeds per plant), total number of full seeds per plant (number of full seeds per plant) and 100-seed weight.

#### CP4-EPSPS protein expression levels in samples

Leaf samples were collected at the vegetative growth stage (V2) [38], flowering stage (R1), podding stage (R3) and mature stage (R7). All samples were quick-frozen with liquid nitrogen and stored at -70 °C for the estimation of EPSPS protein levels, which were determined by ELISA using EPSPS detection kits (Envirologix, Portland, USA). Ten milligrams of each leaf sample as suspended in 1 mL of phosphate-buffered saline containing Tween 20 (PBST buffer), which was supplied as part of the kit, and all procedures were performed according to the manufacturer’s instructions. The absorption was measured on a microplate reader (Infinite M200, Tecan Group Ltd., Männedorf, Switzerland) at 450 nm.

### Statistical analysis

Data are presented as mean ± standard deviation (mean ± sd), One-way analysis of variance (ANOVA) was performed to compare the plant performance differences between different groups, Tukey’s multiple comparison test was used to determine the significance of differences between groups, which were considered significant when *P* < 0.05; all statistical analyses was performed using the SPSS 20.0 software (SPSS Inc.).

### Electronic supplementary material

Below is the link to the electronic supplementary material.

**Additional file 1: Table S1**. Detection of EPSPS by real-time quantitative PCR and EPSPS test strip.

**Additional file 2: Table S2**. Identification of two different genotypes, *EPSPS*-resistant heterozygotes and homozygotes, in F_2_ or F_3_ population plants based on droplet digital PCR.

## Data Availability

The data used in this study are available from the corresponding author on reasonable request.

## Abbreviations

GM: genetically modified
RR: homozygous resistant
RS: heterozygous resistant
SS: homozygous susceptible
