# Supplementary material for: Fitness changes in wild soybean caused by gene flow from genetically modified soybean
Source: BMC Plant Biol. 2023 Sep 14;23:424. doi: 10.1186/s12870-023-04398-2 (PMC10500775; doi:10.1186/s12870-023-04398-2)
Supplement: Supplementary file 1 — Additional file 1: Table S1. Detection of EPSPS by real-time quantitative PCR and EPSPS test strip. [file 12870_2023_4398_MOESM1_ESM.pdf]

Table S1 Detection of EPSPS by real-time quantitative PCR and EPSPS test strip

Material	Ct values of EPSPS gene	Ct values of lectin	Δct	Detection results	EPSPS test strip
F_2-1	20.27	20.81	0.54	P	P
F_2-2	26.85	25.64	1.21	P	P
F_2-3	25.97	25.13	0.84	P	P
F_2-4	Undetermined	25.43		N	N
F_2-5	27.25	27.91	0.66	P	P
F_2-6	25.75	27.38	1.63	P	P
F_2-7	Undetermined	24.19		N	N
F_2-8	26	25.08	0.92	P	P
F_2-9	25.72	25.82	-0.1	P	P
F_2-10	24.45	25.73	1.28	P	P
F_2-11	Undetermined	25.51		N	N
F_2-12	24.31	23.54	0.77	P	P
F_2-13	25.09	26.64	1.55	P	P
F_2-14	25.66	24.87	0.79	P	P
F_2-15	Undetermined	24.89		N	N
F_2-16	20.86	21.89	1.03	P	P
F_2-17	25.35	26.13	0.78	P	P
F_2-18	26.44	26.12	0.32	P	P
F_2-19	25.25	23.68	1.57	P	P
F_2-20	Undetermined	23.98		N	N
F_2-21	26.94	26.44	0.5	P	P
F_2-22	25.34	26.66	1.32	P	P
F_2-23	24.09	25.91	1.82	P	P
F_2-24	Undetermined	24.98		N	N
F_2-25	26.59	25.73	0.86	P	P
F_2-26	24.97	24.94	0.03	P	P
F_2-27	21.02	22.5	1.48	P	P
F_2-28	Undetermined	25.67		N	N
F_2-29	26.76	26.91	0.15	P	P
F_2-30	23.91	26.08	2.17	P	P
F_2-31	24.05	25.46	1.41	P	P
F_2-32	Undetermined	25.32		N	N
F_2-33	26.65	25.14	1.51	P	P
F_2-34	27.77	26.19	1.58	P	P
F_2-35	25.15	24.91	0.24	P	P
F_2-36	25.97	26.84	0.87	P	P
F_2-37	Undetermined	25.18		N	N
F_2-38	23.25	24.79	1.54	P	P
F_2-39	24.88	23.63	1.25	P	P
F_2-40	24.32	25.39	1.07	P	P

F_2-41	24.78	23.26	1.52	P	P
F_2-42	Undetermined	25.17		N	N
F_2-43	22.68	24.52	1.84	P	P
F_2-44	22.29	22.85	0.56	P	P
F_2-45	26.56	25.48	1.08	P	P
F_2-46	26.18	25.01	1.17	P	P
F_2-47	25.21	23.8	1.41	P	P
F_2-48	25.99	26.11	0.12	P	P
F_2-49	Undetermined	24.98		N	N
F_2-50	Undetermined	25.01		N	N
F_2-51	28.92	26.93	1.99	P	P
F_2-52	25.16	27.04	1.88	P	P
F_2-53	28.89	29.86	0.97	P	P
F_2-54	Undetermined	23.93		N	N
F_2-55	27.21	26.65	0.56	P	P
F_2-56	26.72	25.47	1.25	P	P
F_2-57	26.39	25.24	1.15	P	P
F_2-58	Undetermined	24.97		N	N
F_2-59	26.66	26.88	0.22	P	P
F_2-60	26.74	26.69	0.05	P	P
F_2-61	27.2	26.92	0.28	P	P
F_2-62	26.3	26.73	0.43	P	P
F_2-63	Undetermined	25.81		N	N
F_2-64	25.09	25.95	0.86	P	P
F_2-65	25.92	26.05	0.13	P	P
F_2-66	Undetermined	25.31		N	N
F_2-67	25.6	25.74	0.14	P	P
F_2-68	Undetermined	26.01		N	N
F_2-69	26.92	26.13	0.79	P	P
F_2-70	24.63	26.29	1.66	P	P
F_2-71	Undetermined	25.52		N	N
F_2-72	25.36	25.26	0.1	P	P
F_2-73	26.97	26.41	0.56	P	P
F_2-74	25.89	25.81	0.08	P	P
F_2-75	23.97	24.66	0.69	P	P
F_2-76	Undetermined	25.71		N	N
F_2-77	27.62	27.7	0.08	P	P
F_2-78	Undetermined	25.94		N	N
F_2-79	28.07	28.85	0.78	P	P
F_2-80	25.03	27.05	2.02	P	P
F_2-81	22.14	22.38	0.24	P	P
F_2-82	25.52	25.68	0.16	P	P
F_2-83	20.57	20.53	0.04	P	P
F_2-84	25.3	25.61	0.31	P	P

F_2-85	26.03	24.63	1.4	P	P
F_2-86	Undetermined	23.98		N	N
F_2-87	25.59	26.37	0.78	P	P
F_2-88	25.55	23.98	1.57	P	P
F_2-89	23.64	22.79	0.85	P	P
F_2-90	26.09	26.54	0.45	P	P
F_2-91	24.54	24	0.54	P	P
F_2-92	24.8	24.65	0.15	P	P
F_2-93	Undetermined	25.21		N	N
F_2-94	25.59	26.35	0.76	P	P
F_2-95	26.34	26.07	0.27	P	P
F_2-96	27.74	27.4	0.34	P	P
F_2-97	26.43	26.6	0.17	P	P
F_2-98	Undetermined	25.61		N	N
F_2-99	28.12	25.66	2.46	P	P
F_2-100	24.56	26.55	1.99	P	P
F_2-101	Undetermined	25.39		N	N
F_2-102	25.63	25.72	0.09	P	P
F_2-103	24.57	24.91	0.34	P	P
F_2-104	23.36	24.02	0.66	P	P
F_2-105	Undetermined	24.95		N	N
F_2-106	24.85	24.18	0.67	P	P
F_2-107	25.05	25.6	0.55	P	P
F_2-108	24.53	25.17	0.64	P	P
F_2-109	25.75	26.92	1.17	P	P
F_2-110	25.68	25.12	0.56	P	P
F_2-111	26.92	25.79	1.13	P	P
F_2-112	Undetermined	25.76		N	N
F_2-113	26.53	26.74	0.21	P	P
F_2-114	26.33	26.68	0.35	P	P
F_2-115	23.67	24.54	0.87	P	P
F_2-116	Undetermined	24.83		N	N
F_2-117	23.83	24.82	0.99	P	P
F_2-118	23.27	22.67	0.6	P	P
F_2-119	21.81	22.17	0.36	P	P
F_2-120	23.72	24.61	0.89	P	P
F_2-121	22.82	22.39	0.43	P	P
F_2-122	25.86	25.55	0.31	P	P
F_2-123	Undetermined	24.45		N	N
F_2-124	24.79	23.87	0.92	P	P
F_2-125	25.15	26.23	1.08	P	P
F_2-126	26.45	26.88	0.43	P	P
F_2-127	26.58	25.77	0.81	P	P
F_2-128	28.67	27.03	1.64	P	P

F_2-129	26.96	27.43	0.47	P	P
F_2-130	24.05	26.01	1.96	P	P
F_2-131	24.91	24.64	0.27	P	P
F_2-132	25.82	25.76	0.06	P	P
F_2-133	25.7	25.94	0.24	P	P
F_2-134	Undetermined	24.59		N	N
F_2-135	26.6	25.19	1.41	P	P
F_2-136	26.01	25.85	0.16	P	P
F_2-137	28.25	28.3	0.05	P	P
F_2-138	29.11	28.72	0.39	P	P
F_2-139	23.94	25.27	1.33	P	P
F_2-140	23.97	23.35	0.62	P	P
F_2-141	Undetermined	25.19		N	N
F_2-142	25.73	24.83	0.9	P	P
F_2-143	24.67	24.85	0.18	P	P
F_2-144	28.22	28.14	0.08	P	P
F_2-145	26.63	25.13	1.5	P	P
F_2-146	26.4	27.79	1.39	P	P
F_2-147	25.25	25.43	0.18	P	P
F_2-148	25.7	24.43	1.27	P	P
F_2-149	Undetermined	24.94		N	N
F_2-150	25.67	27.17	-1.5	P	P
F_2-151	26.82	25.72	1.1	P	P
F_2-152	Undetermined	23.98		N	N
F_2-153	25.86	25.81	0.05	P	P
F_2-154	24.76	24.86	-0.1	P	P
F_2-155	24.49	26.73	2.24	P	P
F_2-156	24.33	24.75	0.42	P	P
F_2-157	Undetermined	26.74		N	N
F_2-158	24.71	25.14	0.43	P	P
F_2-159	Undetermined	25.97		N	N
F_2-160	25.59	26.03	0.44	P	P
F_2-161	26.25	26.62	0.37	P	P
F_2-162	23.88	24.82	0.94	P	P
F_2-163	25.34	26.75	1.41	P	P
F_2-164	Undetermined	25.13		N	N
F_2-165	23.84	24.88	1.04	P	P
F_2-166	Undetermined	23.42		N	N
F_2-167	24.33	25.19	0.86	P	P
F_2-168	Undetermined	25.18		N	N
F_3-1	20.57	20.7	-0.13	P	P
F_3-2	Undetermined	25.71		N	N
F_3-3	Undetermined	26.43		N	N
F_3-4	26.65	25.33	1.32	P	P

F_3-5	25.98	24.67	1.31	P	P
F_3-6	Undetermined			N	N
F_3-7	25.59	25.69	-0.1	P	P
F_3-8	25.55	24.23	1.32	P	P
F_3-9	24	23.76	0.24	P	P
F_3-10	Undetermined	24.57		N	N
F_3-11	25.29	23.85	1.44	P	P
F_3-12	24.86	24.78	0.08	P	P
F_3-13	21.2	21.62	-0.42	P	P
F_3-14	25.72	26.36	-0.64	P	P
F_3-15	26.33	26.13	0.2	P	P
F_3-16	25.26	24.06	1.2	P	P
F_3-17	25.45	23.95	1.5	P	P
F_3-18	Undetermined	26.39		N	N
F_3-19	24.6	26.97	-2.37	P	P
F_3-20	24.96	26.38	-1.42	P	P
F_3-21	Undetermined	25.46		N	N
F_3-22	29.08	28.46	0.62	P	P
F_3-23	24.66	24.16	0.5	P	P
F_3-24	24.82	24.64	0.18	P	P
F_3-25	Undetermined	26.19		N	N
F_3-26	25	26.06	-1.06	P	P
F_3-27	25.01	26.77	-1.76	P	P
F_3-28	24.67	25.51	-0.84	P	P
F_3-29	25.84	26.69	-0.85	P	P
F_3-30	25.84	24.54	1.3	P	P
F_3-31	26.92	26.2	0.72	P	P
F_3-32	25.09	24.19	0.9	P	P
F_3-33	27.04	26.69	0.35	P	P
F_3-34	26.55	27.37	-0.82	P	P
F_3-35	24.08	25.43	-1.35	P	P
F_3-36	Undetermined	24.93		N	N
F_3-37	24.64	24.47	0.17	P	P
F_3-38	24.05	23.65	0.4	P	P
F_3-39	21.88	23.56	-1.68	P	P
F_3-40	23.61	24.04	-0.43	P	P
F_3-41	22.64	23.58	-0.94	P	P
F_3-42	Undetermined	23.44		N	N
F_3-43	25.68	24.03	1.65	P	P
F_3-44	24.61	22.98	1.63	P	P
F_3-45	24.96	26.66	-1.7	P	P
F_3-46	26.2	26.83	-0.63	P	P
F_3-47	Undetermined	25.76		N	N
F_3-48	27.95	27.43	0.52	P	P

F_3-49	25.88	26.66	-0.78	P	P
F_3-50	29.37	29.57	-0.2	P	P
F_3-51	Undetermined	26.79		N	N
F_3-52	29.28	29.76	-0.48	P	P
F_3-53	28.79	29.57	-0.78	P	P
F_3-54	27.34	29.08	-1.74	P	P
F_3-55	27.22	27.41	-0.19	P	P
F_3-56	26.92	26.1	0.82	P	P
F_3-57	26.77	26.14	0.63	P	P
F_3-58	Undetermined	25.57		N	N
F_3-59	26.59	26.39	0.2	P	P
F_3-60	25.98	24.8	1.18	P	P
F_3-61	25.59	24.51	1.08	P	P
F_3-62	25.99	25.54	0.45	P	P
F_3-63	26.73	27.05	-0.32	P	P
F_3-64	24.88	23.75	1.13	P	P
F_3-65	26.16	26.57	-0.41	P	P
F_3-66	27.07	27.09	-0.02	P	P
F_3-67	Undetermined	24.64		N	N
F_3-68	25.8	25.83	-0.03	P	P
F_3-69	25.41	25.82	-0.41	P	P
F_3-70	26.75	27.11	-0.36	P	P
F_3-71	Undetermined	26.12		N	N
F_3-72	23.53	24.25	-0.72	P	P
F_3-73	25.81	24.65	1.16	P	P
F_3-74	27.58	26.7	0.88	P	P
F_3-75	27.26	26.98	0.28	P	P
F_3-76	27.87	28.77	-0.9	P	P
F_3-77	25.85	26.19	-0.34	P	P
F_3-78	22.12	22.27	-0.15	P	P
F_3-79	25.45	25.6	-0.15	P	P
F_3-80	20.57	21.03	-0.46	P	P
F_3-81	26.48	25.6	0.88	P	P
F_3-82	Undetermined	24.22		N	N
F_3-83	25.47	25.63	-0.16	P	P
F_3-84	25.66	26.74	-1.08	P	P
F_3-85	25.33	25.46	-0.13	P	P
F_3-86	Undetermined	25.18		N	N
F_3-87	26.9	25.61	1.29	P	P
F_3-88	25.31	24.86	0.45	P	P
F_3-89	25.81	24.51	1.3	P	P
F_3-90	20.2	21.83	-1.63	P	P
F_3-91	26.36	24.81	1.55	P	P
F_3-92	25.79	26.16	-0.37	P	P

F_3-93	27.29	26.59	0.7	P	P
F_3-94	25.03	26.09	-1.06	P	P
F_3-95	26.22	26.44	-0.22	P	P
F_3-96	Undetermined	26.21		N	N
F_3-97	Undetermined	26.54		N	N
F_3-98	23.87	24.51	-0.64	P	P
F_3-98	26.07	26.28	-0.21	P	P
F_3-100	24.45	24.72	-0.27	P	P
F_3-101	24.77	25.12	-0.35	P	P
F_3-102	26.98	25.59	1.39	P	P
F_3-103	25.68	25.31	0.37	P	P
F_3-104	Undetermined	23.46		N	N
F_3-105	Undetermined	25.97		N	N
F_3-106	25.37	25.95	-0.58	P	P
F_3-107	24.82	27.39	-2.57	P	P
F_3-108	26.73	26.86	-0.13	P	P
F_3-109	26.04	24.58	1.46	P	P
F_3-110	27.9	28.63	-0.73	P	P
F_3-111	24.34	24.31	0.03	P	P
F_3-112	Undetermined	25.77		N	N
F_3-113	28.21	28.07	0.14	P	P
F_3-114	26.34	26.29	0.05	P	P
F_3-115	27.35	27.14	0.21	P	P
F_3-116	25.46	26.2	-0.74	P	P
F_3-117	25.86	25.81	0.05	P	P
F_3-118	25.54	24.76	0.78	P	P
F_3-119	24.01	24.51	-0.5	P	P
F_3-120	27.11	26.3	0.81	P	P
F_3-121	Undetermined	23.37		N	N
F_3-122	25.13	24.96	0.17	P	P
F_3-123	26.07	24.6	1.47	P	P
GM-1	24.29	23.92	0.37	P	P
GM-2	23.83	25.17	-1.34	P	P
GM-3	20.96	23.34	-2.38	P	P
GM-4	24.29	24.19	0.1	P	P
GM-5	25.77	25.19	0.58	P	P
Wild-1	Undetermined	24.9		N	N
Wild-2	Undetermined	26.93		N	N
Wild-3	Undetermined	27.23		N	N
Wild-4	Undetermined	25.32		N	N
Wild-5	Undetermined	25.25		N	N

Note: Delta Ct was calculated as follows: delta Ct=Ct (target gene)– Ct (*lectin*). *P* positive, *N* negative, *GM* genetically modified, *Wild* wild soybean
