# Supplementary material for: Fitness changes in wild soybean caused by gene flow from genetically modified soybean
Source: BMC Plant Biol. 2023 Sep 14;23:424. doi: 10.1186/s12870-023-04398-2 (PMC10500775; doi:10.1186/s12870-023-04398-2)
Supplement: Supplementary file 2 — Additional file 2: Table S2. Identification of two different genotypes, EPSPS-resistant heterozygotes and homozygotes, in F2 or F3 population plants based on droplet digital PCR. [file 12870_2023_4398_MOESM2_ESM.pdf]

Table S2 Identification of two different genotypes, *EPSPS*-resistant heterozygotes and homozygotes, in F_2 or F_3 population plants based on droplet digital PCR

Material	Gene	Average copy number	Copy number ratio	Genotype
F_2-1	*EPSPS*	289	0.99	RR
	lectin	291.5		
F_2-2	*EPSPS*	183.95	0.94	RR
	lectin	194.8		
F_2-3	*EPSPS*	275	0.56	RS
	lectin	491.5		
F_2-5	*EPSPS*	166	0.52	RS
	lectin	321		
F_2-6	*EPSPS*	138	0.52	RS
	lectin	263		
F_2-8	*EPSPS*	90	1.13	RR
	lectin	79.5		
F_2-9	*EPSPS*	84.5	0.61	RS
	lectin	138		
F_2-10	*EPSPS*	35.7	0.56	RS
	lectin	64		
F_2-12	*EPSPS*	154	0.50	RS
	lectin	311		
F_2-13	*EPSPS*	95.5	0.97	RR
	lectin	98.5		
F_2-14	*EPSPS*	206.5	0.55	RS
	lectin	373.5		
F_2-16	*EPSPS*	319.5	0.62	RS
	lectin	513		
F_2-17	*EPSPS*	234	0.97	RR
	lectin	241.5		

F_2-18	*EPSPS*	240	0.70	RS
	lectin	345		
F_2-19	*EPSPS*	45.8	0.81	RS
	lectin	56.5		
F_2-21	*EPSPS*	298	0.63	RS
	lectin	471.5		
F_2-22	*EPSPS*	320.5	0.94	RR
	lectin	342.5		
F_2-23	*EPSPS*	51	0.68	RS
	lectin	74.5		
F_2-25	*EPSPS*	187	0.51	RS
	lectin	370		
F_2-26	*EPSPS*	125	1.35	RR
	lectin	92.5		
F_2-27	*EPSPS*	178	0.59	RS
	lectin	301		
F_2-29	*EPSPS*	53.5	0.51	RS
	lectin	104.5		
F_2-30	*EPSPS*	158	0.51	RS
	lectin	307.5		
F_2-31	*EPSPS*	58	0.66	RS
	lectin	88		
F_2-33	*EPSPS*	159	0.96	RR
	lectin	165		
F_2-34	*EPSPS*	311	0.65	RS
	lectin	479		
F_2-35	*EPSPS*	228.5	1.16	RR
	lectin	197		
F_2-36	*EPSPS*	279	1.42	RR

	lectin	196		
F_2-38	*EPSPS*	128.5	0.55	RS
	lectin	233.5		
F_2-39	*EPSPS*	117.5	0.58	RS
	lectin	202		
F_2-40	*EPSPS*	335	0.63	RS
	lectin	529.5		
F_2-41	*EPSPS*	32	0.74	RS
	lectin	43		
F_2-43	*EPSPS*	231.5	1.04	RR
	lectin	223		
F_2-44	*EPSPS*	277	0.68	RS
	lectin	408		
F_2-45	*EPSPS*	217.5	0.52	RS
	lectin	418.5		
F_2-46	*EPSPS*	87.5	1.18	RR
	lectin	74		
F_2-47	*EPSPS*	144.5	1.09	RR
	lectin	133		
F_2-48	*EPSPS*	283	0.54	RS
	lectin	527.5		
F_2-51	*EPSPS*	446.5	1.15	RR
	lectin	388.5		
F_2-52	*EPSPS*	48.5	0.52	RS
	lectin	93.5		
F_2-53	*EPSPS*	394	0.56	RS
	lectin	706.5		
F_2-55	*EPSPS*	65.5	0.54	RS
	lectin	121.5		

F_2-56	*EPSPS*	174	0.53	RS
	lectin	331		
F_2-57	*EPSPS*	171	1.12	RR
	lectin	152.5		
F_2-59	*EPSPS*	167	0.53	RS
	lectin	313		
F_2-60	*EPSPS*	295	1.07	RR
	lectin	276.5		
F_2-61	*EPSPS*	167	0.53	RS
	lectin	313		
F_2-62	*EPSPS*	91.5	1.18	RR
	lectin	77.25		
F_2-64	*EPSPS*	135	0.59	RS
	lectin	230.5		
F_2-65	*EPSPS*	24.1	0.57	RS
	lectin	42.5		
F_2-67	*EPSPS*	653	0.95	RR
	lectin	688		
F_2-69	*EPSPS*	70	0.52	RS
	lectin	133.5		
F_2-70	*EPSPS*	273	1.06	RR
	lectin	257		
F_2-72	*EPSPS*	200.5	0.52	RS
	lectin	383.5		
F_2-73	*EPSPS*	586	1.01	RR
	lectin	578.75		
F_2-74	*EPSPS*	345.5	0.64	RS
	lectin	539		
F_2-75	*EPSPS*	177.5	0.52	RS

	lectin	337.5		
F_2-77	*EPSPS*	197.2	1.07	RR
	lectin	184		
F_2-79	*EPSPS*	57.5	0.62	RS
	lectin	93		
F_2-80	*EPSPS*	303.5	1.08	RR
	lectin	280		
F_2-81	*EPSPS*	71.75	1.14	RR
	lectin	63		
F_2-82	*EPSPS*	132.5	0.58	RS
	lectin	229.5		
F_2-83	*EPSPS*	50.7	1.08	RR
	lectin	47		
F_2-84	*EPSPS*	53.25	0.96	RR
	lectin	55.5		
F_2-85	*EPSPS*	62	0.59	RS
	lectin	104.5		
F_2-87	*EPSPS*	190.5	0.65	RS
	lectin	293		
F_2-88	*EPSPS*	164.5	0.88	RR
	lectin	186.5		
F_2-89	*EPSPS*	219.5	0.71	RS
	lectin	310		
F_2-90	*EPSPS*	271	1.02	RR
	lectin	264.5		
F_2-91	*EPSPS*	359	0.55	RS
	lectin	656		
F_2-92	*EPSPS*	242.5	1.12	RR
	lectin	217		

F_2-94	*EPSPS*	128	0.61	RS
	lectin	208.5		
F_2-95	*EPSPS*	246.5	0.56	RS
	lectin	440		
F_2-96	*EPSPS*	142	0.57	RS
	lectin	250.5		
F_2-97	*EPSPS*	351	0.54	RS
	lectin	651		
F_2-99	*EPSPS*	83	0.53	RS
	lectin	158		
F_2-100	*EPSPS*	85.5	0.56	RS
	lectin	152		
F_2-102	*EPSPS*	189.5	1.02	RR
	lectin	186.5		
F_2-103	*EPSPS*	117	0.57	RS
	lectin	206.5		
F_2-104	*EPSPS*	191.5	0.54	RS
	lectin	355.5		
F_2-106	*EPSPS*	69	0.51	RS
	lectin	135		
F_2-107	*EPSPS*	97	0.51	RS
	lectin	188.5		
F_2-108	*EPSPS*	109.5	0.65	RS
	lectin	168.5		
F_2-109	*EPSPS*	32.1	1.07	RR
	lectin	30		
F_2-110	*EPSPS*	235.5	1.08	RR
	lectin	218		
F_2-111	*EPSPS*	185.5	0.65	RS

	lectin	286.5		
F_2-113	*EPSPS*	165	0.55	RS
	lectin	299.5		
F_2-114	*EPSPS*	198.5	0.58	RS
	lectin	341.5		
F_2-115	*EPSPS*	212	0.67	RS
	lectin	318.5		
F_2-117	*EPSPS*	157.5	0.57	RS
	lectin	278.5		
F_2-118	*EPSPS*	40	0.58	RS
	lectin	69.5		
F_2-119	*EPSPS*	259	0.68	RS
	lectin	381.5		
F_2-120	*EPSPS*	511.5	1.03	RR
	lectin	498		
F_2-121	*EPSPS*	191.5	0.60	RS
	lectin	318		
F_2-122	*EPSPS*	304.5	0.54	RS
	lectin	568		
F_2-124	*EPSPS*	349	1.10	RR
	lectin	316		
F_2-125	*EPSPS*	58	0.55	RS
	lectin	105		
F_2-126	*EPSPS*	29.5	0.58	RS
	lectin	50.5		
F_2-127	*EPSPS*	148	0.93	RR
	lectin	159.5		
F_2-128	*EPSPS*	766.5	0.99	RR
	lectin	773		

F_2-129	*EPSPS*	73.5	0.54	RS
	lectin	136		
F_2-130	*EPSPS*	196.5	0.53	RS
	lectin	374		
F_2-131	*EPSPS*	93	0.54	RR
	lectin	171.5		
F_2-132	*EPSPS*	443	1.00	RR
	lectin	444.5		
F_2-133	*EPSPS*	182	0.56	RS
	lectin	325		
F_2-135	*EPSPS*	79.6	0.56	RS
	lectin	141		
F_2-136	*EPSPS*	77	0.53	RS
	lectin	146.5		
F_2-137	*EPSPS*	131.4	1.16	RR
	lectin	113		
F_2-138	*EPSPS*	220.5	0.58	RS
	lectin	383		
F_2-139	*EPSPS*	66.55	1.04	RR
	lectin	64		
F_2-140	*EPSPS*	148.5	0.64	RS
	lectin	231.5		
F_2-142	*EPSPS*	199.5	0.50	RS
	lectin	396		
F_2-143	*EPSPS*	235	0.97	RR
	lectin	242.5		
F_2-144	*EPSPS*	82.5	0.54	RS
	lectin	153		
F_2-145	*EPSPS*	179.5	0.58	RS

	lectin	312		
F_2-146	*EPSPS*	423.5	1.06	RR
	lectin	399.5		
F_2-147	*EPSPS*	117.5	0.54	RS
	lectin	217.5		
F_2-148	*EPSPS*	220.5	0.55	RS
	lectin	403		
F_2-150	*EPSPS*	494	1.05	RR
	lectin	472		
F_2-151	*EPSPS*	341	1.10	RR
	lectin	310		
F_2-153	*EPSPS*	256.5	0.49	RS
	lectin	521.5		
F_2-154	*EPSPS*	217.5	0.54	RS
	lectin	399.5		
F_2-155	*EPSPS*	152.5	0.61	RS
	lectin	248		
F_2-156	*EPSPS*	266.5	0.56	RS
	lectin	475.5		
F_2-158	*EPSPS*	176	0.57	RS
	lectin	309.7		
F_2-160	*EPSPS*	407.5	0.54	RS
	lectin	756.5		
F_2-161	*EPSPS*	49.2	0.94	RR
	lectin	52		
F_2-162	*EPSPS*	97.5	0.52	RS
	lectin	187.5		
F_2-163	*EPSPS*	120	0.57	RS
	lectin	211.5		

F_2-165	*EPSPS*	136	1.13	RR
	lectin	120		
F_2-167	*EPSPS*	192.5	0.51	RS
	lectin	376		
F_3-1	*EPSPS*	376	0.97	RR
	lectin	388.5		
F_3-4	*EPSPS*	237.5	0.58	RS
	lectin	408		
F_3-5	*EPSPS*	273.5	0.60	RS
	lectin	458		
F_3-7	*EPSPS*	506	1.05	RR
	lectin	483.5		
F_3-8	*EPSPS*	511	1.40	RR
	lectin	365.5		
F_3-9	*EPSPS*	579.5	1.23	RR
	lectin	473		
F_3-11	*EPSPS*	270.5	0.55	RS
	lectin	491		
F_3-12	*EPSPS*	333.5	0.53	RS
	lectin	630.5		
F_3-13	*EPSPS*	331.5	0.59	RS
	lectin	564		
F_3-14	*EPSPS*	349.5	0.97	RR
	lectin	359.5		
F_3-15	*EPSPS*	353.5	1.02	RR
	lectin	345		
F_3-16	*EPSPS*	234.5	0.54	RS
	lectin	430.5		
F_3-17	*EPSPS*	255.5	0.63	RS

	lectin	407.5		
F_3-19	*EPSPS*	112.5	0.59	RS
	lectin	190		
F_3-20	*EPSPS*	141	0.53	RS
	lectin	265.5		
F_3-22	*EPSPS*	193	0.99	RR
	lectin	195		
F_3-23	*EPSPS*	282	1.12	RR
	lectin	251		
F_3-24	*EPSPS*	64.5	0.56	RS
	lectin	116		
F_3-26	*EPSPS*	68.5	0.51	RS
	lectin	133.5		
F_3-27	*EPSPS*	343.5	0.98	RR
	lectin	349		
F_3-28	*EPSPS*	230	1.01	RR
	lectin	228.5		
F_3-29	*EPSPS*	114	1.16	RR
	lectin	98.5		
F_3-30	*EPSPS*	68.5	0.58	RS
	lectin	118		
F_3-31	*EPSPS*	134	0.97	RR
	lectin	138		
F_3-32	*EPSPS*	144.5	0.97	RR
	lectin	149.5		
F_3-33	*EPSPS*	38.5	0.51	RS
	lectin	75		
F_3-34	*EPSPS*	157.5	1.01	RR
	lectin	155.5		

F_3-35	*EPSPS*	54.5	1.28	RR
	lectin	42.5		
F_3-37	*EPSPS*	76	1.06	RR
	lectin	71.5		
F_3-38	*EPSPS*	53.5	0.66	RS
	lectin	80.5		
F_3-39	*EPSPS*	116.5	0.61	RS
	lectin	190		
F_3-40	*EPSPS*	175.5	1.06	RR
	lectin	165.5		
F_3-41	*EPSPS*	78	0.60	RS
	lectin	131		
F_3-43	*EPSPS*	251.5	0.52	RS
	lectin	480.5		
F_3-44	*EPSPS*	274	1.04	RR
	lectin	262.5		
F_3-45	*EPSPS*	192.5	0.74	RS
	lectin	261.5		
F_3-46	*EPSPS*	203.5	0.61	RS
	lectin	331		
F_3-48	*EPSPS*	275.5	1.30	RR
	lectin	212.5		
F_3-49	*EPSPS*	279.5	1.35	RR
	lectin	206.5		
F_3-50	*EPSPS*	310	1.30	RR
	lectin	239		
F_3-52	*EPSPS*	332	1.39	RR
	lectin	239.5		
F_3-53	*EPSPS*	163	0.52	RS

	lectin	310.5		
F_3-54	*EPSPS*	143.5	0.58	RS
	lectin	249.5		
F_3-55	*EPSPS*	163.5	0.51	RS
	lectin	318.5		
F_3-56	*EPSPS*	148	0.63	RS
	lectin	233.5		
F_3-57	*EPSPS*	75.5	0.56	RS
	lectin	134		
F_3-59	*EPSPS*	75.5	0.57	RS
	lectin	133		
F_3-60	*EPSPS*	48.5	0.60	RS
	lectin	81.5		
F_3-61	*EPSPS*	61.5	0.55	RS
	lectin	111.5		
F_3-62	*EPSPS*	155	1.37	RR
	lectin	113.5		
F_3-63	*EPSPS*	154.5	1.32	RR
	lectin	117		
F_3-64	*EPSPS*	55	0.59	RS
	lectin	94		
F_3-65	*EPSPS*	56	0.56	RS
	lectin	99.5		
F_3-66	*EPSPS*	313.5	0.92	RR
	lectin	339		
F_3-68	*EPSPS*	302.5	1.15	RR
	lectin	263.5		
F_3-69	*EPSPS*	266.5	0.51	RS
	lectin	521.5		

F_3-70	*EPSPS*	259	0.60	RS
	lectin	434.5		
F_3-72	*EPSPS*	50	0.60	RS
	lectin	84		
F_3-73	*EPSPS*	70	0.64	RS
	lectin	109.5		
F_3-74	*EPSPS*	301.5	0.94	RR
	lectin	321		
F_3-75	*EPSPS*	272.5	1.27	RR
	lectin	214.5		
F_3-76	*EPSPS*	263	0.52	RS
	lectin	507.5		
F_3-77	*EPSPS*	265	0.54	RS
	lectin	490		
F_3-78	*EPSPS*	179.5	1.20	RR
	lectin	150		
F_3-79	*EPSPS*	182.5	0.98	RR
	lectin	186		
F_3-80	*EPSPS*	170	0.55	RS
	lectin	309.5		
F_3-81	*EPSPS*	183	0.76	RS
	lectin	240.5		
F_3-83	*EPSPS*	32.5	0.64	RS
	lectin	50.5		
F_3-84	*EPSPS*	134.5	0.57	RS
	lectin	238		
F_3-85	*EPSPS*	42.5	0.52	RS
	lectin	81.5		
F_3-87	*EPSPS*	43	0.70	RS

	lectin	61.5		
F_3-88	*EPSPS*	84.5	0.53	RS
	lectin	158		
F_3-89	*EPSPS*	74.5	0.55	RS
	lectin	136		
F_3-90	*EPSPS*	159.5	0.68	RS
	lectin	234.5		
F_3-91	*EPSPS*	125.5	1.00	RR
	lectin	126		
F_3-92	*EPSPS*	316	0.55	RS
	lectin	575.5		
F_3-93	*EPSPS*	296	1.32	RR
	lectin	223.5		
F_3-94	*EPSPS*	154.5	0.52	RS
	lectin	297		
F_3-95	*EPSPS*	169	0.55	RS
	lectin	306		
F_3-98	*EPSPS*	319.5	0.58	RS
	lectin	554.5		
F_3-99	*EPSPS*	335.5	0.62	RS
	lectin	537.5		
F_3-100	*EPSPS*	49	0.53	RS
	lectin	93		
F_3-101	*EPSPS*	128	0.57	RS
	lectin	224.5		
F_3-102	*EPSPS*	143	1.04	RR
	lectin	137.5		
F_3-103	*EPSPS*	55	1.00	RR
	lectin	55		

F_3-106	*EPSPS*	160	0.54	RS
	lectin	297		
F_3-107	*EPSPS*	179.5	0.60	RS
	lectin	297		
F_3-108	*EPSPS*	292.5	0.98	
	lectin	299		
F_3-109	*EPSPS*	236	0.63	RS
	lectin	375.5		
F_3-110	*EPSPS*	57.5	0.55	RS
		105		
F_3-111	*EPSPS*	28.5	0.51	RS
	lectin	56		
F_3-113	*EPSPS*	376.5	1.00	RR
	lectin	377		
F_3-114	*EPSPS*	383.5	1.25	RR
	lectin	307.5		
F_3-115	*EPSPS*	56.5	0.56	RS
	lectin	100.5		
F_3-116	*EPSPS*	141	0.60	RS
	lectin	234.5		
F_3-117	*EPSPS*	123	0.52	RS
	lectin	235		
F_3-118	*EPSPS*	155	1.17	RR
	lectin	133		
F_3-119	*EPSPS*	150.5	0.60	RS
	lectin	252.5		
F_3-120	*EPSPS*	29	0.62	RS
	lectin	47		
F_3-122	*EPSPS*	124.5	0.68	RS

	lectin	183.5		
F_3-123	*EPSPS*	142.5	0.59	RS
	lectin	239.5		
Wild-1	*EPSPS*	0	0	SS
	lectin	343		
Wild-2	*EPSPS*	0	0	SS
	lectin	356		
Wild-3	*EPSPS*	0	0	SS
	lectin	289.5		

Note: *RR* homozygous resistant, *RS* heterozygous resistant, *SS* homozygous susceptible.
